# RETRACTION: Echinacoside Protects Against 6-Hydroxydopamine-Induced Mitochondrial Dysfunction and Inflammatory Responses in PC12 Cells via Reducing ROS Production

**DOI:** 10.1155/ecam/9891208

**Published:** 2025-08-06

**Authors:** Evidence-Based Complementary and Alternative Medicine

RETRACTION: Y.-H. Wang, Z.-H. Xuan, S. Tian, and G.-H. Du, “Echinacoside Protects Against 6-Hydroxydopamine-Induced Mitochondrial Dysfunction and Inflammatory Responses in PC12 Cells via Reducing ROS Production,” *Evidence-Based Complementary and Alternative Medicine* 2015 (2015): 189239, https://doi.org/10.1155/2015/189239.

The above article, published online on 18 February 2015 in Wiley Online Library (https://wileyonlinelibrary.com), has been retracted by John Wiley & Sons Ltd.

The retraction has been agreed upon following an investigation into concerns raised by a third party, which identified overlap with another article published by the same author group.

Unexpected similarity has been identified between the PC-12 cells in Figures 2(a) and 2(e), and Figures 1(a) and 1(e) in a previous publication [1], where they are described as the same cell type with different experimental conditions. In addition, unexpected similarities were found between the graph shown in Figures 4C(e) and 5(a) of [1].

As a result, the conclusions of the current article are considered unreliable.

The authors have been informed of the retraction but did not provide a response.
